# Influence of Cross-Linkers on the Wash Resistance of Chitosan-Functionalized Polyester Fabrics

**DOI:** 10.3390/polym16162365

**Published:** 2024-08-21

**Authors:** Tanja Pušić, Tea Bušac, Julija Volmajer Valh

**Affiliations:** 1 Faculty of Textile Technology, University of Zagreb, Prilaz Baruna Filipovića 28a, 10000 Zagreb, Croatia; tanja.pusic@ttf.unizg.hr (T.P.); tea.busac@ttf.unizg.hr (T.B.); 2 Faculty of Mechanical Engineering, University of Maribor, Smetanova 17, 2000 Maribor, Slovenia

**Keywords:** polyester, functionalization, chitosan, cross-linkers, stability, washing

## Abstract

This study investigates the wash resistance of polyester fabrics functionalized with chitosan, a biopolymer known for its biocompatibility, non-toxicity, biodegradability and environmentally friendly properties. The interaction of chitosan with synthetic polymers, such as polyester, often requires surface treatment due to the weak natural affinity between the two materials. To improve the interaction and stability of chitosan on polyester, alkaline hydrolysis of the polyester fabric was used as a surface treatment method. The effectiveness of using cross-linking agents 1,2,3,4-butane tetracarboxylic acid (BTCA) and hydroxyethyl methacrylate (HEMA) in combination with ammonium persulphate (APS) to improve the stability of chitosan on polyester during washing was investigated. The wash resistance of polyester fabrics functionalized with chitosan was tested after 1, 5 and 10 washes with a standard ECE detergent. Staining tests were carried out to evaluate the retention of chitosan on the fabric. The results showed that polyester fabrics functionalized with chitosan without cross-linkers exhibited better wash resistance than the fabrics treated with crosslinkers.

## 1. Introduction

After cellulose, chitosan is the most abundant biopolymer [1] and is used as a natural polysaccharide with exceptional biological and physico-chemical properties, as well as its environmental friendliness, in various areas [2] such as medicine, biomedicine [3], pharmacy, cosmetics, the textile, chemical and paper industries and agriculture. Chitosan has two important structural parameters, namely the degree of deacetylation (DD) and the molecular weight (MW). The degree of deacetylation determines the solubility and biodegradability of chitosan, which is closely related to the degree of crystallization [1,4,5].

Chitosan has primary amino groups with a pKa value of 6.3, the presence of which determines the properties of chitosan and its pH sensitivity. At a pH value below its pKa, chitosan is a polycation, while at a pH value ≤ 4, it is completely protonated. On the other hand, chitosan loses its electrical charge due to the deprotonation of the amine groups and becomes insoluble when the pH rises above 6. Therefore, the chitosan is prepared in an acidic medium consisting of inorganic and organic acids, preferably with organic acids [5,6,7].

Chitosan is of increasing interest when it comes to adding functionalities to textile surfaces [8]. However, the weak interaction of chitosan to textile fibers is the main problem in its application. Citric acid and other weak oxidizing agents have been shown to promote effective cross-linking between chitosan and textile substrates such as cotton and its blends [6,8,9,10,11].

The compatibility of two polymers in a mixture can be extended by modification depending on the composition [12]. Considering the partial compatibility of biopolymer chitosan with polyester as a synthetic polymer, modification of the materials should be carried out by classical or advanced methods to introduce functional groups [8,13,14,15].

The alkaline hydrolysis of polyester textiles with sodium hydroxide hydrolyzes the ester groups in the polymer chain so that modified fragments remain in the polyester structure. Alkaline-hydrolyzed polyesters have an improved reactivity and hydrophilicity of the textiles due to the introduction of carboxyl groups (-COOH) [8,14]. The reaction of alkaline hydrolysis can be catalyzed by cationic promoters, which can reduce the concentration of sodium hydroxide or short reaction time [11,13,16]. Alkaline hydrolysis reduces the tensile properties of polyester textiles, but functionalization with chitosan restores them and improves resistance to deformation, the wetting ability and hydrophilicity, antimicrobial properties, reduces charging by static electricity and reduces the proportion of released fragments from polyester textiles in the washing process [11,17].

Efficacy and durability of chitosan-functionalized polyester fabrics are certain limitations, so the aim of this study was to analyze the stability of three chitosan-functionalized polyester fabrics (chitosan itself, chitosan with BTCA and chitosan with HEMA/APS) during the washing process. The solution of chitosan (low molecular weight, LMW) was prepared in hydrochloric acid as a pH-controlling agent, which differs from the majority of available protocols referring to acetic acid [6,8,14,18]. Furthermore, the functionalization of polyester fabrics was carried out with a chitosan concentration of 1%, which is less than most protocols in the selected published papers [7,8,19,20]. The interaction of chitosan as well as chitosan cross-linkers with polyester was improved by alkaline hydrolysis of the polyester reference fabric samples. The effect of the functionalization of a polyester reference fabric with a biopolymer chitosan before and after one, three and five washing cycles was analyzed by a staining test with acid dyestuff. The staining test is a simple, effective and affordable method to confirm the presence of chitosan on fabric and does not require expensive research equipment.

## 2. Materials and Methods

### 2.1. Materials and Reagents

The research was carried out on the polyester reference fabric, supplied by Centre for Testmaterials, CFT, Vlaardingen, The Netherlands, specified by mass per unit area of 156 g/m^2^, density in warp direction 27.7 cm^−1^ and 20 cm^−1^ threads in weft direction, fineness of the warp threads 30.4 tex and fineness of weft threads 31.9 tex. The ultrasonic cutter model TTS400, Sonowave S.r.l., Legnano MI, Italy was used to prepare 30 × 50 cm polyester reference fabric samples.

The following chemicals were used: sodium hydroxide (NaOH), supplied by Ivero d.d.; chitosane (low molecular weight (LMW) with an 85% degree of deacetylation); 1,2,3,4-butanetetracarboxylic acid (BTCA) (99%) and calcium carbonate (CaCO_3_), supplied by Sigma Aldrich, St. Louis, USA; hydrochloric acid (HCl, 37%), supplied by GRAM-MOL, Zagreb, Croatia, 2-hydroxyethyl methacrylate (HEMA) (97%) and 4-methoxyphenol, supplied by Thermo Fisher (Kandel) GmbH, Karlsruhe, Germany; ammonium persulphate (APS), supplied by BDH Chemicals, Leuven, Belgium; standard ECE A detergent from SDL Atlas; and dye Telon^®^ Blue M-GLW (C.I. Acid Blue 221) supplied by DyStar Colours Distribution GmbH, Raunheim, Germany.

### 2.2. Procedures

#### 2.2.1. Alkali Hydrolysis

In order to improve the interaction between the surface of the synthetic polymer and the biopolymer chitosan, the polyester reference fabric was modified by treatments in solutions of NaOH. Alkaline hydrolysis of the fabric was carried out in solutions of 10 g/L (1AH), 20 g/L (2AH) and 30 g/L NaOH (3AH) with a bath ratio of 1:5. The polyester fabrics were inserted in a chamber of a total volume of 10 L, sodium hydroxide of a certain concentration prepared in soft water (50 ppm CaCO_3_) was added and the hydrolysis started at 20 °C in a Polymat laboratory apparatus, Mathis, Switzerland. The chamber was heated by gradient of 4 °C/min until a temperature of 98 °C was achieved. The duration of alkali hydrolysis was 30 min. After the end of alkaline hydrolysis, the polyester fabrics were removed from the bath and rinsed four times due to removal of residual alkali, two times at 80 °C and two times with cold water. The so-prepared samples were air-dried.

#### 2.2.2. Functionalization of Polyester Fabrics with Chitosan

The low-molecular-weight chitosan with a deacetylation degree of 85% was used to functionalize the reference polyester fabrics. The multiphase preparation of the stable chitosan solution (1%) is pH-sensitive and time-consuming. Hydrochloric acid (1 mol/L) was used to prepare the acidic chitosan solution with a pH value of 3.62 and to adjust the pH value after stirring overnight.

For the functionalization of the polyester reference fabric, the cross-linker BTCA was added to the prepared chitosan solution (1%) in amount of 5% per mass of chitosan. The HEMA stabilized with approximately 500 ppm 4-methoxyphenol in an amount of 5% and a catalyst APS in an amount of 1%, were added to the chitosan solution (1%) before functionalization of the polyester reference fabric.

The application of chitosan on polyester fabrics was carried out in three variants: with chitosan itself (Ch), chitosan with cross-linker 1,2,3,4-butanetetracarboxylic acid (Ch*BTCA) and chitosan with cross-linker 2-hydroxyethyl methacrylate with ammonium persulphate (Ch*HEMA/APS).

The functionalization of the reference polyester fabric without (U) and after the alkaline hydrolysis processes (1AH, 2AH, 3AH) with chitosan (Ch) solution, chitosan with BTCA (Ch*BTCA) solution and chitosan with HEMA/APS (Ch*HEMA/APS) solution was carried out on the padder and stenter, Ernst Benz, Rümlang-Zurich, Switzerland, at a pressure of 12.5 kg/cm. Thermal processes, drying at 90 °C for 40 s and curing at 130 °C for 20 s, followed the functionalization of the polyester fabrics.

#### 2.2.3. Washing Process

The stability of chitosan-polyester fabrics in the washing process was tested according to HRN EN ISO 6330 (2A) procedure [21] using a standard ECE A detergent (1.25 g/L) at 60 °C with a bath ratio of 1:7 over 1, 3 and 5 cycles in the Rotawash laboratory machine, Atlas SDL. Washing in hard water was followed by rinsing over 4 cycles with a bath ratio of 1:8 and air-drying in a horizontal position. The labelling of the treatments and samples is shown in Table 1.

#### 2.2.4. Staining Test

The effect of the functionalization of a polyester reference fabric with a biopolymer chitosan in three variations (with chitosan itself, and with two cross-linkers (BTCA, HEMA/APS), and its stability in the washing process was analyzed by a staining test carried out using the dye Telon^®^ Blue M-GLW (C.I. Acid Blue 221). The 5 × 5 cm samples were soaked in Petri dishes with the dye solution (1 %) for 15 min. After soaking, the samples were rinsed with water to remove excess non-fixed dye and air-dried.

**Scheme 1:**
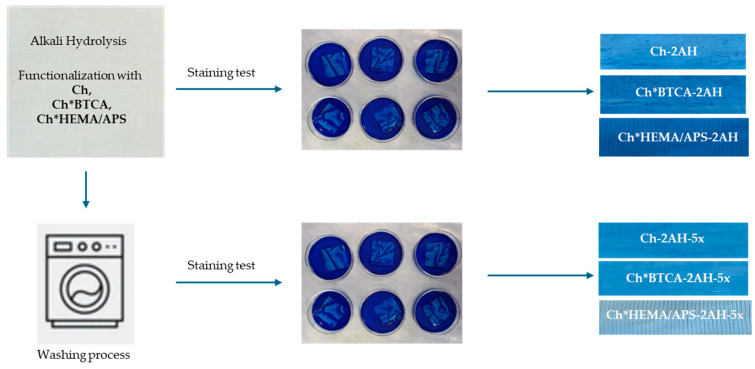
Schematic representation of processes.

All processes are presented in Scheme 1.

### 2.3. Methods

#### 2.3.1. Gravimetric Analysis

Samples of the reference polyester fabrics before and after alkaline hydrolysis were weighed to determine the weight loss (∆m) as an indicator of the reaction with a surface and topochemical effect of alkaline hydrolysis.

#### 2.3.2. Streaming Potential Method

The zeta potential of samples was determined by streaming potential method in the SurPASS electrokinetic analyzer equipped with a titration unit and an adjustable gap cell (AGC) controlled with Attract software 2.0 (all from Anton Paar GmbH, Graz, Austria). After stabilization of the measurement parameters in the electrokinetic analyzer, the streaming potential of the polyester fabric samples before and after alkaline hydrolysis in 20 g/L NaOH (sample 2AH) in the electrolyte solution (1 mM/L KCl) was measured as a function of pH, starting from alkaline (adjusted with 1 mol/L NaOH) to acidic (adjusted with 1 mol/L HCl). During the titration procedure, the streaming potential in mV and other parameters were recorded, from which the zeta potential was calculated as a function of pH according to the Helmholtz–Smoluchovsky equation [22,23].

#### 2.3.3. Microscopic Observation

Photographs of stained polyester fabrics (micrographs) were taken using a DinoLite digital microscope, Premier IDCP B.V., Almere, The Netherlands, at 50× magnification.

#### 2.3.4. Remission Spectrophotometry

The spectral values of polyester reference fabrics were determined using the DataColor SF300 spectrophotometer, aperture 2.2 cm, illumination D65 and geometry d/8°.

The whiteness of untreated and alkaline hydrolyzed polyester reference fabrics was evaluated according to AATCC test method 110 [24].

In accordance with [25], the total color difference, the Δ*E*, of all dyed samples was calculated as follows (Equation (1)):

(1) $$DE^{*}=\sqrt{({DL}^{*}{)}^{2}+({DC}^{*}{)}^{2}+({DH}^{*}{)}^{2}}$$

where

DL*—difference in lightness (DL* = L*washed sample − L*standard),
  DC*—difference in chroma (DC* = C*washed sample − C*standard),
  DH*—difference in hue (DH* = H*washed sample − H*standard).

Additionally, the evaluation of the change in color of washed samples to the appropriate standard based on greyscale values was evaluated according to ISO and AATCC [26,27].

Color strength (K/S value) was determined for selected chitosan-functionalized polyester samples before and after 5 washes.

## 3. Results

### 3.1. Alkaline Hydrolysis

The alkaline hydrolysis of polyester cleaved its ether bonds and formed -COOH groups, which increased its reactivity and hydrophilicity. The increase in polar -COOH groups after alkaline hydrolysis improved the wettability of polyester. However, this treatment also created craters that changed the porosity of the material and caused a reduction in mass.

#### 3.1.1. Weight Loss

The weight losses of polyester reference fabrics resulting from the performed alkaline hydrolysis processes are presented in Table 2.

The alkaline hydrolysis of polyester textiles is a topochemical process in sodium hydroxide solution at high temperatures, which should be maintained so that the weight loss does not exceed 5% [8]. The weight loss of polyester fibers brings with it many advantageous properties, such as increased absorbency, hydrophilicity, moisture recovery and dye absorption, while at the same time reducing the tendency to pilling and the generation of static charge.

The results in Table 2 show the effect of NaOH concentration on the weight loss of the polyester fabrics. Treatment with 30 g/L (3AH) resulted in a weight loss of 7.1%, which, according to [8], exceeds recommended value of 5%. Despite this fact, all polyester reference fabric samples (1AH, 2AH, 3AH) were further functionalized with chitosan solutions to analyze the influence of the surface modification on the uptake of the biopolymer chitosan as well as driven by cross-linkers applied in functionalization of polyester fabric.

#### 3.1.2. Whiteness Quality

As the whiteness quality is an important criterion in all phases of pre-treatment, the whiteness degree (W_CIE_), the basic whiteness (Y) and the change in tint (TV, TD) of the untreated (U) and the alkaline-hydrolyzed polyester samples (AH) were measured accordingly, as shown in Table 3.

The results in Table 3 show changes in whiteness quality that were not completely harmonized. Namely, the 1AH process reduced the whiteness by 10 units, with some reduction in the basic whiteness of (Y) and a deviation in tint (R1). The 2AH and 3AH processes, on the other hand, increased the degree of whiteness by 3–4 units. Enhanced whiteness quality proves a higher degree of alkaline cleaning and removal of preparations on the surface of the polyester reference fabric [28].

#### 3.1.3. Streaming Potential

**Figure 1:**
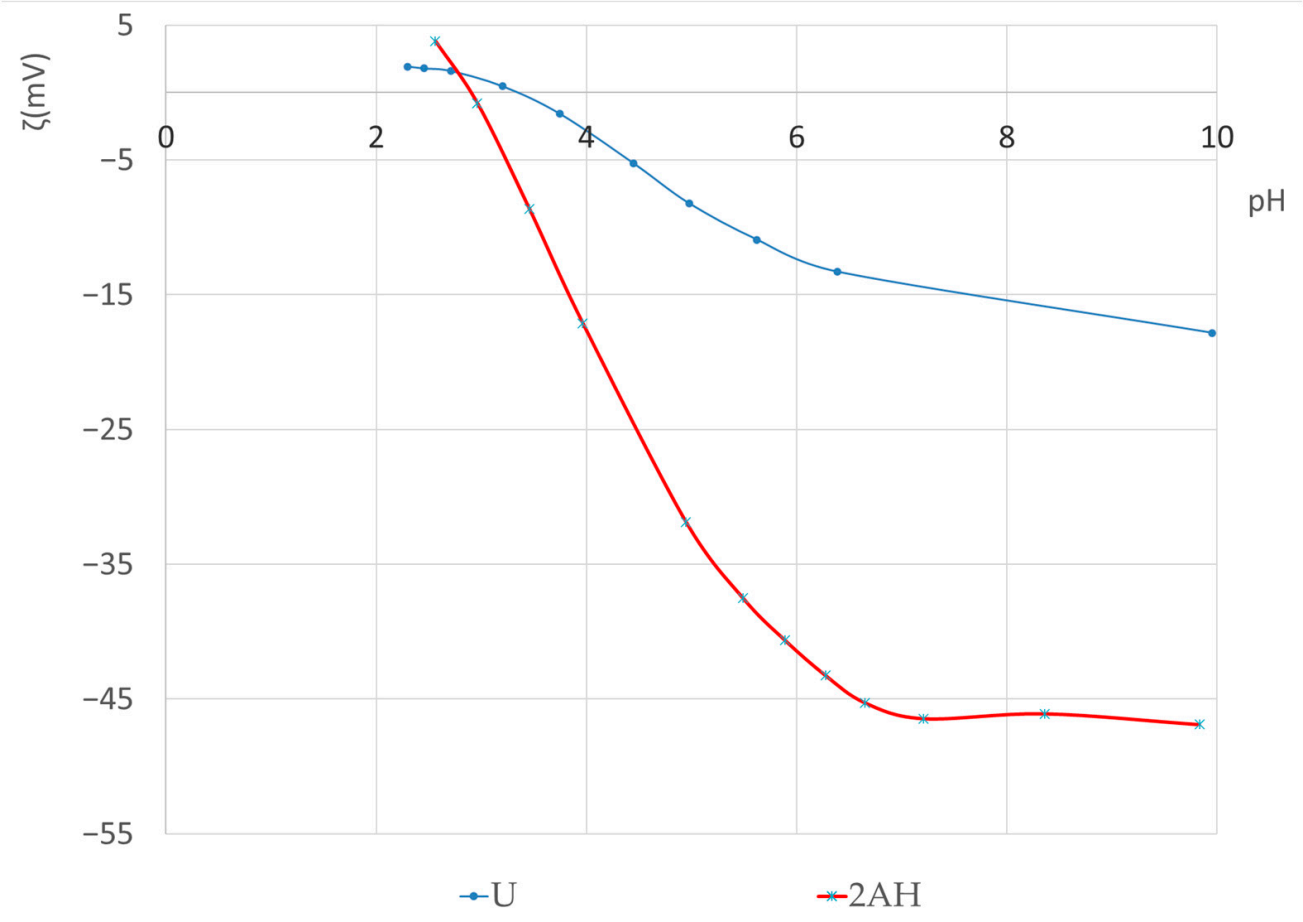
Zeta potential of polyester fabric before (U) and after (2AH) alkaline hydrolysis in variation of pH.

The level of surface modification of polyester fabric after alkaline hydrolysis was assessed by the streaming potential method. The comparison of the zeta potential curves of untreated (U) and alkali-treated polyester fabric (2AH) (Figure 1) proved a modification of the surface. More negative values of the zeta potential showed that the alkali-treated polyester fabric had more accessible groups.

### 3.2. Stainability of Chitosan-Functionalized Polyester Fabrics

The staining test is a practical method for detecting the presence of chitosan. Depending on the substrate, different types of dyes (reactive, disperse, acid) can be used that react specifically with chitosan. Color intensity confirms how much chitosan is present on the surface or deposited from the surface. Both samples were colored blue, with the blank sample having a light color and the chitosan-functionalized polyester sample having a dark color [8,20,28,29]. According to [14], chitosan improves the stainability of the fabrics with acid dye, Telon Turquoise (C.I. Acid Blue 185), owing to the ionic interaction between protonated amino groups and sulfonic groups of the dye ions.

The color intensity and homogeneity of chitosan-functionalized polyester fabrics was tested by staining with Telon^®^ Blue M-GLW (C.I. Acid Blue 221).

Untreated and alkaline hydrolyzed samples were also stained with the Telon Blue dyestuff, and the appearance of untreated and alkaline treated samples is shown in Table 4.

The differences between the samples in Table 4 indicate a different dye substantivity depending on the topography and the surface. The pale blue color of the untreated samples was according to the literature [29]. The colors of 1AH, 2AH and 3AH were consistent with the whiteness and roughness influenced by the topochemical reaction that was the result of alkaline hydrolysis. The alkaline hydrolysis impaired the binding of Telon^®^ Blue M-GLW to the polyester samples.

In Table 5, the microscopic images of cthe olored polyester reference samples (without and after the alkaline hydrolysis processes) functionalized with chitosan, chitosan with BTCA and chitosan with HEMA/APS before and after 1, 3 and 5 wash cycles are shown.

The presence of chitosan on the surface of the polyester reference samples was detected by the blue coloration of the samples before and after washing (Table 5). Alkaline hydrolysis of polyester fabric improved its functionalization with chitosan by increasing the availability of reactive sites on the fiber surface. The treatment involved breaking ester linkages within the polyester polymer, leading to the formation of carboxyl (-COOH) and hydroxyl (-OH) groups on the fabric surface. These functional groups provided active sites for the bonding of chitosan, which was rich in amino groups (-NH₂). The degree of functionalization with chitosan was most pronounced when the polyester fabric was treated with a 30 g/L NaOH solution. According to this coloring of the samples in Table 5, the chitosan was preserved on surface of all washed samples.

An indicator for the stability of chitosan in the washing process are the spectral values and the fastness degree according to AATCC and ISO A05, shown in Table 6, Table 7, Table 8, Table 9 and Table 10.

The results in Table 6, Table 7, Table 8 and Table 9 show that the wash cycles influenced the spectral values of the polyester reference fabrics. Just the first wash cycle reduced the spectral values, which continue to change over three cycles. The spectral values of all samples treated with chitosan were slightly reduced at five cycles compared to three cycles. The most favorable spectral values, confirming the good interaction of chitosan with polyester, were obtained from the chitosan-functionalized samples (Ch) and the chitosan alkaline-hydrolyzed samples (Ch-3AH). According to these indicators, it is desirable to carry out the alkaline hydrolysis of (3AH) as a preparatory phase of the topographical changes that favor compatibility with chitosan, as shown in Table 9.

Micrographs of untreated (U) and alkaline-hydrolyzed (1AH, 2AH, 3AH) polyester reference fabrics functionalized with chitosan and cross-linker BTCA before and after 1, 3 and 5 washes are shown in Table 10.

Microscopic images of colored Ch*BTCA polyester reference samples (U, 1AH, 2AH, 3AH) showed uneven coloration on the surface of all chitosan treated samples. BTCA, as a cross-linker during processing, influenced the localization of chitosan on the surface of the polyester reference fabric. The degree of this non-uniformity in coloration was particularly pronounced in the alkaline-hydrolyzed samples treated with chitosan and BTCA (Ch*BTCA-1AH, Ch*BTCA-2AH, Ch*BTCA-3AH) when compared to the untreated chitosan BTCA sample (Ch*BTCA-U). The non-uniform fragments were deposited during washing processes. The change in the intensity of the blue color showed that five washing cycles caused the deposition of chitosan from the surface of the polyester reference fabrics. The quantification of the differences between the samples before and after the washes is carried out using the spectral values and the color fastness evaluation in Table 11, Table 12, Table 13 and Table 14.

The changes in the spectral values in Table 11, Table 12, Table 13 and Table 14 show that the stability of chitosan with the cross-linker Ch*BTCA in the washing process was weaker than that of Ch itself. The differences in the spectral values in Table 11, Table 12, Table 13 and Table 14 show the influence of the sodium hydroxide concentration and the washing process on the deposition of chitosan. The change in the spectral values of the samples evaluated using the ISO and AATCC greyscale values showed a lower deposition of chitosan from sample Ch*BTCA-2AH in the washing process. According to all spectral parameters, alkali hydrolysis at 20 g/L was the optimum preparation concentration for good and stable chitosan uptake on polyester fabrics.

Micrographs of untreated (U) and alkaline-hydrolyzed (1AH, 2AH, 3AH) polyester reference fabric functionalized with chitosan and cross-linker HEMA/APS before and after 1, 3 and 5 washes are shown in Table 15.

The intensity of the blue color of polyester standard fabrics functionalized with crosslinker HEMA/APS was slightly lighter than that of chitosan with cross-linker BTCA. However, the blue shade of all samples was retained over five wash cycles. The structural elements (warp and weft threads) of all samples washed with Ch*HEMA/APS were more visible compared to the samples washed with Ch and Ch*BTCA. This proves that the chitosan had almost completely separated from the surface of the Ch*HEMA/APS samples during the washing process.

The quantification of the changes in the samples functionalized with chitosan and the crosslinker HEMA/APS through the wash cycles is shown in Table 16, Table 17, Table 18 and Table 19.

The changes in the spectral values in Table 16, Table 17, Table 18 and Table 19 show that the stability of chitosan with the cross-linker HEMA/APS in the washing process was weaker than that of Ch itself and Ch*BTCA. Already after the first wash cycle, the persistence of color fastness was rated as 1, which confirms the further hydrolysis of fabrics treated with Ch*HEMA/APS in alkaline detergent solution. The spectral value, the total color difference (DE) was such that all samples were graded by the same category of changes, grade 1, although the lightness changes (DL*), chroma changes (DC*) and hue changes (DH*) of the samples differed. Within the analyzed series of chitosan-treated samples, Ch*HEMA/APS and Ch*HEMA/APS-1AH retained more chitosan than Ch*HEMA/APS-2AH and Ch*HEMA/APS-3AH.

Taking into account the less use of chemicals and optimal effects, primary parameters weight loss and degree of whiteness, the alkaline hydrolysis (2AH) proved to be optimal. So, the results of the color strength (K/S) for all samples (2AH, Ch-2AH, Ch*BTCA-2AH, Ch*HEMA/APS-2AH) before and after five wash cycles (Ch-2AH-5x, Ch*BTCA-2AH-5x, Ch*HEMA/APS-2AH-5x) were selected for the final stability checking point, as shown in Table 20.

The results in Table 20 confirm the presence of chitosan on chitosan-functionalized polyester fabrics according to color strength and blue coloration; HEMA/APS provided the best results. However, stability analysis over five wash cycles gave priority to the treatment of polyester fabric with chitosan itself, without cross-linkers.

## 4. Conclusions

The chitosan solution, which was prepared by adding hydrochloric acid as a pH-sensitizing solution, confirmed the applicability of this acid in all variations, with and without cross-linking agents, due to its viscosity and time stability. Alkaline hydrolysis of the polyester fabric improved the degree of its functionalization with the biopolymer chitosan. Within the applied sodium hydroxide concentration, the optimal chitosan-functionalized polyester was obtained with 2% NaOH. The cross-linking agents 1,2,3,4-butanetetracarboxylic acid (BTCA) and hydroxyethyl methacrylate (HEMA) with ammonium persulphate (APS) used did not improve the degree of functionality of polyester and alkaline-hydrolyzed polyester with chitosan. The results confirmed the influence of the washing process on the reduction of the color strength of the chitosan-functionalized polyester, the chitosan-functionalized, alkaline-hydrolyzed polyester and, consequently, on the deposition of chitosan. The staining test for all polyester fabric samples before and after the washing process confirmed that the cross-linkers used did not improve the stability of the chitosan-functionalized polyester.

The staining test confirmed that five washing cycles had an impact on the reduction of spectral values, as quantified by the values of total color difference, fastness levels and color strength compared to unwashed polyester fabrics.

## Figures and Tables

**Table 1:** Designation of polyester fabrics.

Polyester Sample	Label
Untreated	U
Alkali treated (NaOH) 10 g/L, 20 g/L, 30 g/L	1AH, 2AH, 3AH
Chitosan treated	Ch-U Ch-1AH, Ch-2AH, Ch-3AH
Chitosan treated with BTCA	Ch*BTCA-U Ch*BTCA-1AH, Ch*BTCA-2AH, Ch*BTCA-3AH
Chitosan treated with HEMA/APS	Ch*HEMA/APS-U Ch*HEMA/APS-1AH, Ch*HEMA/APS-2AH, Ch*HEMA/APS-3AH
Washed, cycles	−1×, −3×,−5×

**Table 2:** Weight loss of polyester reference fabrics after alkaline hydrolysis.

Reference Polyester Fabric	∆m (%)
1AH	1.5 ± 0.04
2AH	4.2 ± 0.06
3AH	7.1 ± 0.01

**Table 3:** Whiteness degree of untreated and the alkaline-hydrolyzed polyester samples.

Samples	W	TV	TD	Y
U	68.1	0.1		81.8
1AH	56.7	−0.5	R1	80.3
2AH	72.2	0.0		82.2
3AH	71.6	0.0		81.7

**Table 4:** Stained untreated and alkaline-hydrolyzed samples.

Stained Sample	U	1AH	2AH	3AH
Micrograph	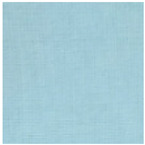	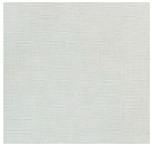	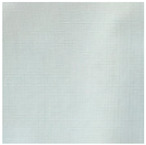	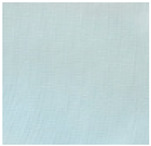

**Table 5:** Micrographs of stained polyester samples (U, 1AH, 2AH, 3AH) functionalized with chitosan (Ch) before and after 1, 3 and 5 washing cycles.

Samples	Ch-U	Ch-1AH	Ch-2AH	Ch-3AH
Micrograph	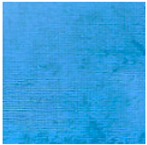	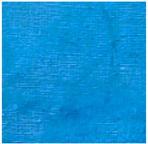	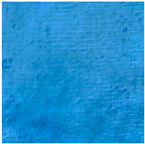	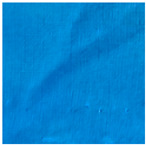
Samples	Ch-U-1x	Ch-1AH-1x	Ch-2AH-1x	Ch-3AH-1x
Micrograph	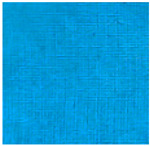	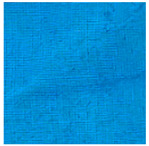	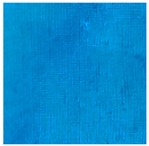	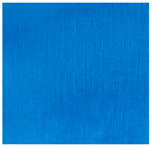
Samples	Ch-U-3x	Ch-1AH-3x	Ch-2AH-3x	Ch-3AH-3x
Micrograph	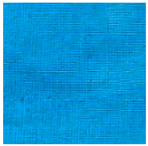	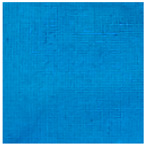	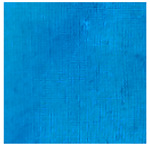	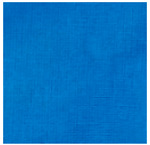
Samples	Ch-U-5x	Ch-1AH-5x	Ch-2AH-5x	Ch-3AH-5x
Micrograph	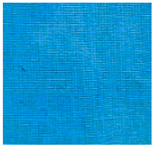	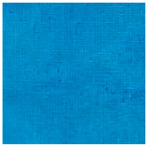	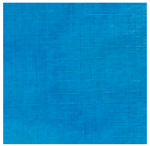	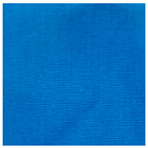

**Table 6:** Spectral values of the washed chitosan polyester reference fabric, Ch-U.

Standard Ch-U	**Cycles**	**ISO A05**	**AATCC**	**DE**	DL*	DC*	DH*
	1	4	3-4	2.564	1.318	1.553	−1.258
	3	3-4	3	3.813	−0.041	3.461	−0.251
	5	3-4	3	2.986	2.344	1.068	−0.864

**Table 7:** Spectral values of washed chitosan alkaline-hydrolyzed polyester reference fabric, Ch-1AH.

Standard Ch-1AH	**Cycles**	**ISO A05**	**AATCC**	**DE**	DL*	DC*	DH*
	1	3-4	3	3.780	0.919	3.234	−1.292
	3	3-4	3	3.778	0.738	3.503	−0.829
	5	3-4	3	3.590	2.352	2.200	−1.156

**Table 8:** Spectral values of washed chitosan alkaline-hydrolyzed polyester reference fabric, Ch-2AH.

Standard Ch-2AH	**Cycles**	**ISO A05**	**AATCC**	**DE**	DL*	DC*	DH*
	1	2-3	2	6.466	5.728	−1.235	−2.715
	3	3	3	3.979	2.988	1.982	−1.130
	5	2	2	7.207	6.639	−0.999	−2.548

**Table 9:** Spectral values of washed chitosan alkaline-hydrolyzed polyester reference fabric, Ch-3AH.

Standard Ch-3AH	**Cycles**	**ISO A05**	**AATCC**	**DE**	DL*	DC*	DH*
	1	4	3	3.302	–0.683	3.029	0.695
	3	4	4	2.118	1.359	1.311	0.280
	5	3-4	3-4	2.837	1.825	1.851	0.080

**Table 10:** Micrographs of stained polyester samples (U, 1AH, 2Ah, 3AH) with chitosan and cross-linker BTCA (Ch*BTCA) before and after 1, 3 and 5 washes.

Samples	Ch*BTCA-U	Ch*BTCA-1AH	Ch*BTCA-2AH	Ch*BTCA-3AH
Micrograph	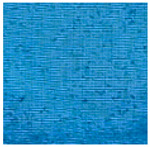	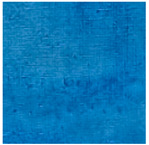	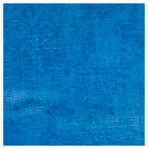	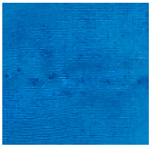
Samples	Ch*BTCA-U-1x	Ch*BTCA-1AH-1x	Ch*BTCA-2AH-1x	Ch*BTCA-3AH-1x
Micrograph	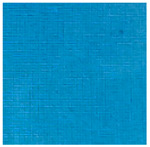	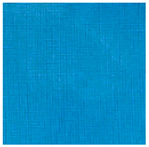	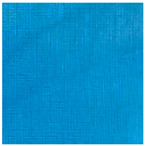	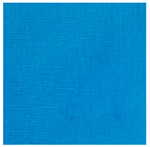
Samples	Ch*BTCA-U-3x	Ch*BTCA-1AH-3x	Ch*BTCA-2AH-3x	Ch*BTCA-3AH-3x
Micrograph	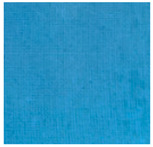	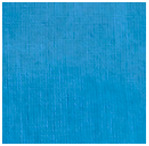	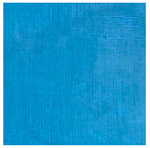	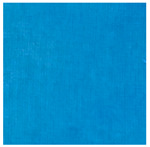
Samples	Ch*BTCA-U-5x	Ch*BTCA-1AH-5x	Ch*BTCA-2AH-5x	Ch*BTCA-3AH-5x
Micrograph	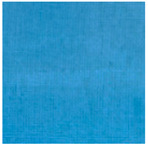	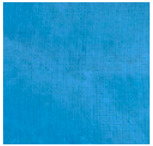	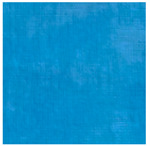	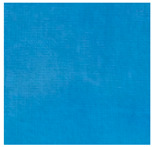

**Table 11:** Spectral values of the washed chitosan polyester reference fabric, Ch*BTCA.

Standard Ch*BTCA	**Cycles**	**ISO A05**	**AATCC**	**DE**	DL*	DC*	DH*
	1	2-3	2-3	4.665	3.973	0.077	−2.274
	3	2	2	6.321	5.744	−1.445	−2.189
	5	2-3	2-3	4.938	4.588	−0.648	−1.686

**Table 12:** Spectral values of the washed chitosan alkaline-hydrolyzed polyester reference fabric, Ch*BTCA-1AH.

Standard Ch*BTCA-1AH	**Cycles**	**ISO A05**	**AATCC**	**DE**	DL*	DC*	DH*
	1	2	2	6.338	5.512	0.502	−3.072
	3	2	2	7.217	6.581	−0.633	−2.764
	5	2	2	8.241	7.479	−1.491	−3.090

**Table 13:** Spectral values of washed chitosan alkaline-hydrolyzed polyester reference fabric, Ch*BTCA-2AH.

Standard Ch*BTCA-2AH	**Cycles**	**ISO A05**	**AATCC**	**DE**	DL*	DC*	DH*
	1	3	3	3.923	1.958	2.665	−1.722
	3	2-3	2-3	5.261	4.627	0.159	−2.418
	5	2-3	2-3	4.673	3.715	1.541	−2.262

**Table 14:** Spectral values of washed chitosan alkaline-hydrolyzed polyester reference fabric, Ch*BTCA-3AH.

Standard Ch*BTCA-3AH	**Cycles**	**ISO A05**	**AATCC**	**DE**	DL*	DC*	DH*
	1	2	2	6.749	5.811	0.089	−3.410
	3	2	1-2	8.526	7.465	−1.447	−3.840
	5	1-2	1-2	9.487	8.407	−2.075	−3.870

**Table 15:** Micrographs of stained polyester samples (U, 1AH, 2Ah, 3AH) with chitosan and cross-linker HEMA/APS (Ch*HEMA/APS) before and after 1, 3 and 5 washes.

Samples	Ch*HEMA/APS-U	Ch*HEMA/APS-1AH	Ch*HEMA/APS-2AH	Ch*HEMA/APS-3AH
Micrograph	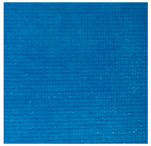	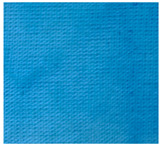	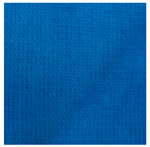	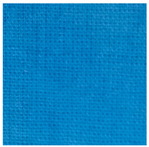
Samples	Ch*HEMA/APS-U-1x	Ch*HEMA/APS-1AH-1x	Ch*HEMA/APS-2AH-1x	Ch*HEMA/APS-3AH-1x
Micrograph	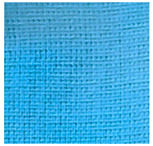	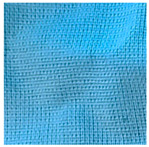	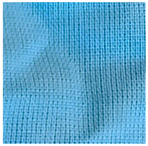	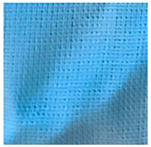
Samples	Ch*HEMA/APS-U-3x	Ch*HEMA/APS-1AH-3x	Ch*HEMA/APS-2AH-3x	Ch*HEMA/APS-3AH-3x
Micrograph	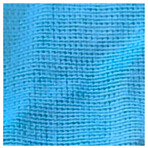	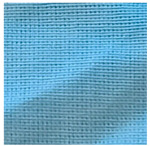	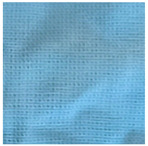	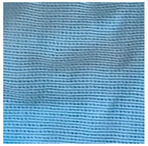
Samples	Ch*HEMA/APS-U-5x	Ch*HEMA/APS-1AH-5x	Ch*HEMA/APS-2AH-5x	Ch*HEMA/APS-3AH-5x
Micrograph	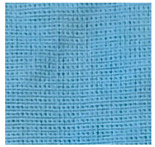	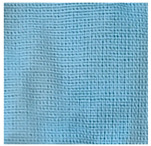	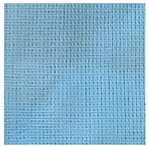	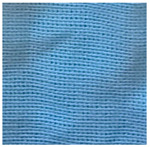

**Table 16:** Spectral values of the washed chitosan polyester reference fabric, Ch*HEMA/APS-U.

Standard Ch*HEMA/APS-U	**Cycles**	**ISO A05**	**AATCC**	**DE**	DL*	DC*	DH*
	1	1	1	22.514	15.027	−14.805	−7.866
	3	1	1	22.023	14.794	−14.536	−7.379
	5	1	1	22.588	15.803	−14.250	−7.573

**Table 17:** Spectral values of the washed chitosan alkaline-hydrolyzed polyester reference fabric, Ch*HEMA/APS-1AH.

Standard Ch*BTCA-1AH	**Cycles**	**ISO A05**	**AATCC**	**DE**	DL*	DC*	DH*
	1	1	1	17.446	11.939	−11.246	−5.672
	3	1	1	23.543	17.406	−15.008	−6.460
	5	1	1	25.545	18.144	−16.470	−7.187

**Table 18:** Spectral values of the washed chitosan alkaline-hydrolyzed polyester reference fabric, Ch*HEMA/APS-2AH.

Standard Ch*BTCA-2AH	**Cycles**	**ISO A05**	**AATCC**	**DE**	DL*	DC*	DH*
	1	1	1	31.000	22.942	−18.379	−9.937
	3	1	1	33.907	25.103	−20.304	−10.360
	5	1	1	35.050	25.540	−21.510	−10.651

**Table 19:** Spectral values of the washed chitosan alkaline-hydrolyzed polyester reference fabric, Ch*HEMA/APS-3AH.

Standard Ch*BTCA-3AH	**Cycles**	**ISO A05**	**AATCC**	**DE**	DL*	DC*	DH*
	1	1	1	26.368	19.758	−14.951	−9.007
	3	1	1	30.535	22.440	−18.457	9.387
	5	1	1	31.422	22.929	−19.159	−9.720

**Table 20:** Color strength (K/S) of 2AH and chitosan-functionalized polyester samples before and after 5 wash cycles.

Samples	K/S	K/S, Checksum	Micrographs
2AH	0.02	0.69	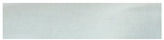
Ch-2AH	0.24	63.88	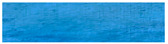
Ch-2AH-5x	0.13	42.34	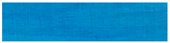
Ch*BTCA-2AH	0.21	56.71	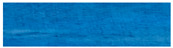
Ch*BTCA-2AH-5x	0.13	40.95	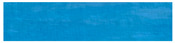
Ch*HEMA/APS-2AH	0.21	89.67	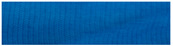
Ch*HEMA/APS-2AH-5x	0.12	32.49	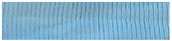

## Data Availability

The original contributions presented in the study are included in the article; further inquiries can be directed to the corresponding author.
